# Robust Detection of Cancer Markers in Human Serums Using All-Dielectric Metasurface Biosensors

**DOI:** 10.3390/bios13030377

**Published:** 2023-03-13

**Authors:** Masanobu Iwanaga

**Affiliations:** National Institute for Materials Science (NIMS), 1-1 Namiki, Tsukuba 305-0044, Japan; iwanaga.masanobu@nims.go.jp

**Keywords:** cancer marker, PSA, CEA, sandwich assay, human serum, metasurface, fluorescence detection, robustness

## Abstract

One of the most significant characteristics, which biosensors are supposed to satisfy, is robustness against abundant molecules coexisting with target biomolecules. In clinical diagnoses and biosensing, blood, plasma, and serum are used daily as samples. In this study, we conducted a series of experiments to examine the robustness of all-dielectric metasurface biosensors, which comprise pairs of a highly fluorescence-enhancing silicon nanopellet array and a transparent microfluidic chip. The metasurface biosensors were shown to have high performance in detecting various targets from nucleic acids to proteins, such as antigens and antibodies. The present results show almost four-order wide dynamic ranges from 0.16 ng/mL to 1 μg/mL for prostate-specific antigen (PSA) and from 2 pg/mL to 25 ng/mL for carcinoembryonic antigen (CEA). The ranges include clinical criteria for PSA, 4 ng/mL and CEA, 5 ng/mL. To date, a systematic demonstration of robustness has not been reported regarding the metasurface biosensors. In detecting cancer markers of PSA and CEA in human serums, we demonstrate that the metasurface biosensors are robust enough in a wide target concentrations, including the clinical diagnosis criteria.

## 1. Introduction

The detections of biomolecules is a crucial part of clinical diagnosis and health monitoring. To detect target biomolecules in the practical situations, we need not only high sensitivity, but also robustness against other biomolecules present in abundance. It is widely known that human blood usually comprises 55% blood cells, such as red corpuscles and platelets, and 45% plasma, which has proteins, such as immunoglobulin G (IgG) and albumin, other glycerin, and inorganic salts. Normally, concentrations of globulin and albumin in blood are 20–35 and 35–45 g/L, respectively [1]. For medical diagnosis, cancer markers, such as prostate specific antigen (PSA) and carcinoembryonic antigen (CEA), are examined in a range around 5 ng/mL [2]. Thus, biomarker molecules are much rarer than abundant biomolecules; for example, IgG concentration, which is normally 8.61–17.47 mg/mL [2], is $10^{6}$-fold higher than the clinical criteria of PSA and CEA. Therefore, robustness is inevitably a requirement for practical biosensors.

**Figure 1:**
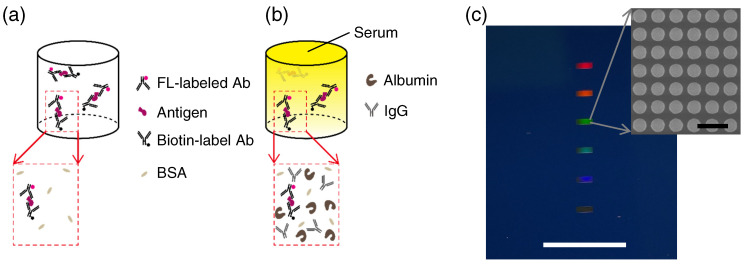
Key concepts of this study. (**a**) Target antigens in a purified condition. (**b**) Targets in a human serum where abundant impeding molecules, exist together with a small number of targets. Antibody (Ab) labeled with fluorescence (FL) molecule (magenta dot), antigen (purple), Ab labeled with biotin (black dot), and bovine serum albumin (BSA, light brown) are shown. Additionally, albumin (brown) and immunoglobulin G (IgG, Y-shaped) are schematically illustrated. (**c**) Photograph (color) of an all-dielectric metasurface substrate and top-view scanning-electron-microscopy image (gray scale), providing a magnified view. The metasurface was a 300-nm periodic array of silicon nanopellets. Six metasurface areas of small rectangular shapes (2.1×0.7 mm${}^{2}$ each) in the photo were designed to correspond to six microfluidic channels. White and black scale bars indicate 10 mm and 500 nm, respectively.

Human serums are most commonly examined for cancer markers in clinical examinations [3,4,5]. Accordingly, biosensors applicable to cancer markers were often tested for their robustness using human serums [6,7,8,9,10,11,12,13,14,15,16,17]. All-dielectric metasurface biosensors [18,19,20,21] and plasmon–photon hybrid metasurface biosensors [22] were recently reported as efficient fluorescence (FL) biosensors. Figure 1a,b illustrates two situations where sandwich complexes of antibody (Ab)—antigen—Ab are formed. Complexes in the former are in a buffer suitable for proteins, whereas complexes in the latter are in a human serum that contains abundant biomolecules, such as albumin and IgG. For immobilization of the complexes, one of the Abs is labeled with biotin, and for FL detection, the other Ab is labeled with FL molecules. The biotin-labeled Abs serve as capture Abs and the FL-labeled Abs work as detection Abs in an ordinary immunoassay scheme, e.g., enzyme-linked immunosorbent assay (ELISA). Here, we report a series of proof-of-concept experiments using the all-dielectric metasurface biosensors for the detection of cancer markers that coexist with other actual proteins, such as albumin, IgG, and different cancer markers, thereby clarifying the robustness of metasurface biosensors.

A color photograph of an all-dielectric metasurface substrate is shown in Figure 1c, where six metasurface areas of small rectangular shapes appear, exhibiting diffraction colors. A white scale bar indicates 10 mm. An scanning-electron-microscopy (SEM) image, which magnifies a metasurface area, is shown on a gray scale. The SEM image was taken in a top-view manner, presenting a periodic array of circular silicon nanopellets of 200 nm height. The periodic length was 300 nm and the diameter of the silicon nanopellets was 224±4 nm. A black scale bar represents 500 nm.

## 2. Materials and Methods

### 2.1. All-Dielectric Metasurface Biosensors

The all-dielectric metasurface substrates were fabricated through the nanolithography process for silicon-on-insulator (SOI) substrates. The SOI substrates comprised a top layer of crystalline silicon of 200 nm thickness, a middle layer of buried oxide (or SiO${}_{2}$) of 375 nm thickness, and a base silicon wafer of 675 μm thickness. Following the nanopatterns written on an electron-beam resist, only the top layer was normally etched down to the middle layer, which resulted in silicon nanopellet arrays (Figure 1c). The top-down nanolithography process has been previously reported in detail [18,23]. Originally, the all-dielectric metasurfaces were conceived with being stimulated from the finding of large FL-enhancing effects in plasmon–photon hybrid metasurfaces [24,25,26]. We note that the metasurface substrates are reusable after the washdown of the adhered proteins in experiments. The washdown was conducted in two steps: (i) the substrates were first washed for 5 min in a neutral ultrasonic cleaning liquid (7-5337-02, As-One, Osaka, Japan), which was 10-times diluted in advance using purified water, were rinsed four times in the purified water under applying ultrasonic waves (5 min each), and were made dry blowing N${}_{2}$ gas; (ii) the substrates were immersed in so-called piranha solution, which consists of 96% sulfuric acid and 30% H${}_{2}$O${}_{2}$ liquid (the volume ratio 3:1), for 15 min, and were rinsed with distillated water for 20 min. The validity of the piranha solution to remove organic deposits on silicon nanostructures was previously reported [27]. After the washdown, we did not find any residue emitting FL on the substrates. This reusability has the potential to reduce running costs significantly.

To control the flow of liquid reagents including human serums, we combined the metasurface substrate with a microfluidic (MF) chip and prepared a metasurface biosensor. The MF chip was made of polydimethylsiloxane (PDMS), which was transparent to visible light and was designed to have six MF paths in accordance with the six metasurface areas (Figure 1c). Inlet and outlet holes were introduced at both ends of each MF path to facilitate connecting from the outside; this can be seen in previous reports [20,21]. The PDMS was naturally absorbed on the metasurface substrate, enabling us to flow the liquid reagents.

### 2.2. Biomolecules and Reagents

Target cancer markers were purchased from companies. We used native human PSA protein (ab78528, Abcam, Cambridge, UK) and CEA (CEA15-N-100, Alpha Diagnostic, San Antonio, TX, USA) as the targets. In diluting the targets to specific concentrations, a sample diluent, NS buffer (ab193972, Abcam), was applied, consisting of phosphate-buffer saline (PBS) and bovine serum albumin. Human serum pool (12181201, Cosmo Bio, Tokyo, Japan), which was a mixture of 10-person serums free from specific diseases, was used for testing the robustness of the metasurface biosensors.

The sandwich complex of Ab—antigen—Ab was designed to have labels, such as biotin-Ab—antigen—Ab-FL. In accordance with this design, a FL label, HiLyte Flour 555 (HL555), was conjugated, in advance, to the Abs using a labeling kit (LK14, Dojindo Laboratories, Kumamoto, Japan). Additionally, biotin was conjugated using a labeling kit (LK03, Dojindo Laboratories). Abs for the PSA and CEA used in this study were mouse monoclonal, being reactive to human PSA and CEA, respectively. Anti-PSA Ab (8A9B8, GenScript, Nanjing, China) and anti-CEA Ab (ab4451, Abcam) were biotin-conjugated, and anti-PSA Ab (MAB6729, Abnova, Taipei, Taiwan) and anti-CEA Ab (10-2370, Fitzgerald Industries, Acton, MA, USA) were HL555-conjugated. After the conjugations, the Abs were collected nominally at 0.5 mg/mL, and then the concentrations of the labeled Abs were tested by light absorption measurement. The HL555-labeling ratios can be evaluated from the light absorbance; we found that HL555 molecules:anti-PSA Ab ≈ 4:1 and HL555 molecules:anti-CEA Ab ≈ 7:1. For the collection and dilutions of the labeled Abs, PBS at pH 7.4 (164-25511, FujiFilm Wako Pure Chemicals, Osaka, Japan) was used. To immobilize the sandwich complexes on the metasurfaces, Cys-streptavidin (Cys-SA, PRO1005, ClickBiosystems, Richardson, TX, USA) was employed, which can bind to the outermost surface of silicon nanopellets and effectively capture the biotin-labeled sandwich complexes.

### 2.3. MF Protocols and FL Detection

MF protocol for PSA detection was as follows. PBS was first used to fill the MF paths. Second, the Cys-SA solution, adjusted to 20 μg/mL using the PBS, was flowed at 10–11 μL/min for 10 min on the metasurface areas. In a previous experiment measuring sensorgram for the Cys-SA [22], the immobilized amount reached the maximum at approximately 8 min, so that we set the time to flow the Cys-SA to be 10 min. The Cys-SA was rinsed with the PBS for 7 min; then, a background FL image was captured for 2 s on each channel under illumination by a green LED (M530F2, Thorlab, Newton, NJ, USA). The FL images were acquired using an uncooled CCD camera (Infinity3S-1URC, Teledyne-Lumenera, Ottawa, Canada). Subsequently, the biotin-labeled anti-PSA Abs of 2.0 μg/mL were flowed at 10–11 μL/min for 10 min on the metasurface areas, and then they were rinsed for 7 min with the PBS. The target PSA proteins diluted with the NS buffer or the human serum were flowed at approximately 8 μL/min for 20 min, and then they were rinsed for 7 min with the PBS. Due to the low concentrations, the PSA was flowed at the low flow rate. The HL555-labeled Abs were flowed at 10–11 μL/min for 10 min, and then the final rinse was conducted at 19–20 μL/min for 8 min with PBS-Tween20 (PBS-T, 163-24361, FujiFilm Wako Pure Chemicals), pH 7.4. Following the MF-flow protocol just above, the green LED light illuminated on each channel, and each FL image was acquired for 2 s exposure time to detect the PSA. A custom-build software was used to control MF flows, liquid reagent changes, and the FL measurements in sequence. The automated setup was as compact as 40×30×60 cm${}^{3}$.

MF protocol for CEA detection differed from that for PSA. The sandwich complexes were incubated independently of the MF-flow system because we found that the step flows for the PSA were not suitable for CEA. This difference probably comes from smaller affinity between the CEA and Abs compared to that between PSA and the Abs. The target CEA was adjusted to particular concentrations for each experiment, typically, 0.04–25 ng/mL using the NS buffer or the human serum diluent (serum: NS buffer =1:4 in volume). For the serum, the target CEA was first spiked in the human serum pool and the concentration was 200 μg/mL in the human serum. Afterwards, the target was diluted using the human serum diluent. The anti-CEA Abs were diluted to 10 μg/mL for the serum-diluted CEA and to 2 μg/mL for the NS-buffer diluted CEA using the PBS. Typically, the 50 μL CEA and the two 100 μL anti-CEA Abs were mixed and incubated at 299 K for 40 min at 400 rpm in the dark. After the incubation, the test liquid was flowed at 10–11 μL/min for 23 min on the metasurfaces that was already covered with the Cys-SA; then, the MF paths were rinsed with the PBS-T at 19–20 μL/min for 5 min. Subsequently, FL imaging was conducted on each channel for 3 s exposure time. When the FL images were analyzed, we used a free software, ImageJ [28].

## 3. Results

### 3.1. Detections of Individual Cancer Markers

#### 3.1.1. PSA

**Figure 2:**
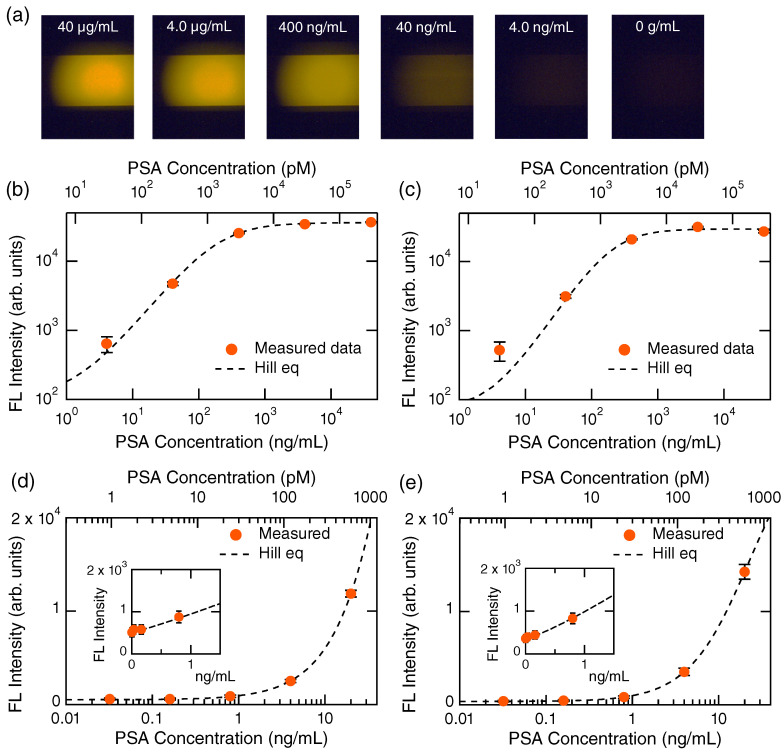
PSA detection. (**a**) FL images at high PSA concentrations from 40 μg/mL to 0 g/mL (from left to right, respectively). The target PSA was diluted using the sample diluent buffer. (**b**) and (**c**) Detection curves of high-concentration PSA diluted with the sample diluent buffer and human serums, respectively. These are presented on a log-log scale. Dashed curves represent fitted curves by the Hill equation (Equation (1)). (**d**,**e**) Detection curves of PSA diluted with the sample diluent buffer and human serums, respectively, presented on a semi-log scale. Dashed curves are fitted curves using the Hill equation. The target concentrations were in a range from 4.0 ng/mL to 0 g/mL. Insets magnify a range near 0 g/mL, presented on a linear scale.

Figure 2 shows a series of experimental results regarding PSA detection. In Figure 2a, FL images at high PSA concentrations from 40 μg/mL to 0 g/mL are presented from left to right, respectively. The all-dielectric metasurfaces are located near the center of the images; bright horizontal areas are explicitly seen at 40, 4.0, and 0.4 μg/mL; the metasurfaces at the other concentrations are placed similarly though it is not seen brightly. FL intensities were most intense at the center of the excitation LED spots, which take a broad Gaussian shape and can be seen explicitly at 40 and 4.0 μg/mL. The FL intensities were quantified with setting and analyzing a circular region around the center in common with the six MF channels.

The FL intensities in Figure 2a were quantified in the circular regions around the center of excitation spots and plotted in Figure 2b using orange closed circles with error bars on a log-log scale. The error bars were evaluated using Gaussian fitting, being standard deviation σ of the FL-intensity distributions. The detection profile was fitted using Hill equation [29], which is mathematically equivalent to the so-called four-parameter logistic equation:

(1) $$y=y_{0}+\left(S-y_{0}\right)\frac{x^{n}}{x^{n}+K_{D}^{n}}$$

where *y* denotes the FL intensity, $y_{0}$ is the zero level without any target, *S* is the saturation FL intensity that is regarded as a proportional constant in fitting, *x* is the concentration of target, *n* is the degree of cooperative reaction, and $K_{D}$ is the dissociation constant [30,31]. In the MF paths, stable liquid flow rates are maintained; therefore, the immobilization process on the metasurface is an equilibrium chemical reaction, which is described using the Hill equation (Equation (1)). From the fitted results, it was determined that S=36148.3, n=1.18, $K_{D}=195.2$ ng/mL, and $y_{0}=110.0$ in Figure 2b. When the fitted value of *n* is more than 1, it is suggested that the reaction is cooperative [32]. Therefore, the fitted value *n* indicates that immobilization of detection Abs with the FL label occurred in a cooperative manner. The value $y_{0}$ indicates a zero level in the FL measurement, being approximately six times smaller than the FL intensity at 4.0 ng/mL, which is currently the clinical criterion value for PSA [2]. Figure 2b shows that even 100-fold higher PSA concentrations can be detected by the metasurface biosensors in a scaled manner. The parameter $K_{D}$ denotes the dissociation constant and indicates the target concentration at the half height of the Hill curve.

The PSA in a human serum was successfully detected, as shown in Figure 2c. The detection profile is quite similar to that in Figure 2b. This result indicates that PSA detection using the metasurface biosensor is robust, even in human serums. The fitting parameters in Figure 2c were S=29620.3, n=1.41, $K_{D}=206.9$ ng/mL, and $y_{0}=72.2$. As a result, the binding reaction is evaluated by *n*, suggesting that the cooperative reaction is similar to that without human serums. In addition, the interplay of PSA and the Abs is not affected by human serums.

Figure 2d,e shows the FL detection results in a PSA concentration range lower than that in Figure 2b,c, respectively, and is presented on a semi-log scale; insets magnify a range near 0 ng/mL in a linear scale. Orange closed circles with error bars denote measured data, and dashed curves are fitted curves using the Hill equation (Equation (1)). The measured data are well-reproduced using the Hill equation and are scaled. We here define dynamic range, such as a range where we can discriminate measured signals more than 1σ and read out concentrations using a reasonable scale (e.g., linear or the Hill curve). The dynamic range of the PSA detection in the step-flow protocol is found to be 0.16–1000 ng/mL, which is almost four orders of concentrations. In the semi-log plots, it is difficult to see the changes in FL intensity at low concentrations below 1 ng/mL; however, the linear plots in the insets exhibit the scaled responses of the metasurface biosensors, even at the range that is 25-fold smaller than the clinical criterion. We refer to that of the dynamic range, which is mainly limited by the performance of the uncooled CCD camera; indeed, a confocal FL microscopy enabled us to access much lower concentrations when we detected the spike proteins of SARS-CoV-2 [21].

#### 3.1.2. CEA

**Figure 3:**
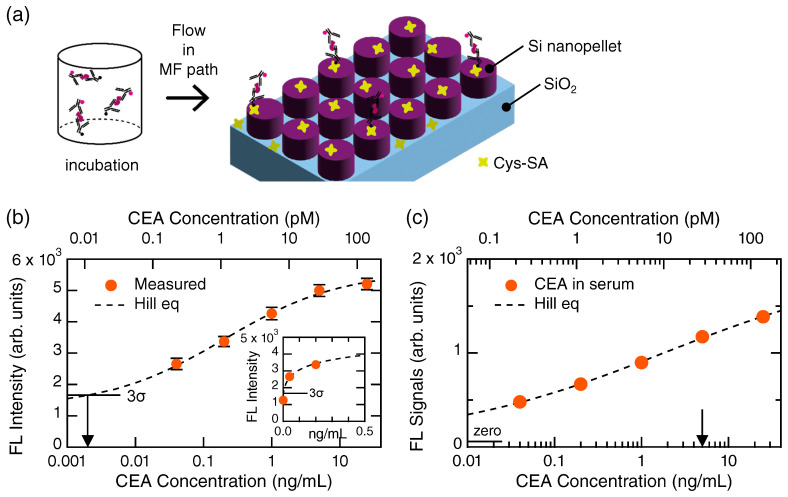
CEA detection. (**a**) Schematic of incubation and immobilization of the CEA sandwich bodies on the metasurface of a periodic array of Si nanopellets. (**b**) Detection of CEA in the sample diluent buffer. Measured FL intensities are shown using orange closed circles with error bars. Dashed curves denote fitted curves by the Hill equation (Equation (1)). 3σ level from the zero level is indicated by a horizontal bar; the crossing point with the Hill curve means the limit of detection, indicating 0.002 ng/mL (arrow). The inset magnifies the detection curve around the zero concentration on a linear scale. (**c**) FL signals from CEA in the human serum. Horizontal bar indicates zero-signal level. Arrow indicates the clinical criterion of 5 ng/mL.

Figure 3a illustrates a protocol from incubation to immobilization of the CEA-sandwich bodies on the metasurface biosensor composed of a periodic array of silicon nanopellets, though the MF path around the periodic array is not drawn. The binding molecules, Cys-SA, were immobilized in advance on the silicon nanopellets. After the immobilization of the Cys-SA and the rinse of unbound molecules, the CEA-sandwich bodies flowed in the MF paths and effectively bound via the biotin–avidin interplay on the silicon nanopellets. Unbound CEA-sandwich bodies were rinsed with the PBS-T. Afterwards, the FL imaging was conducted from the top of metasurface biosensor; the optical configuration of the biosensor has been described in previous publications [20,21].

**Figure A1:**
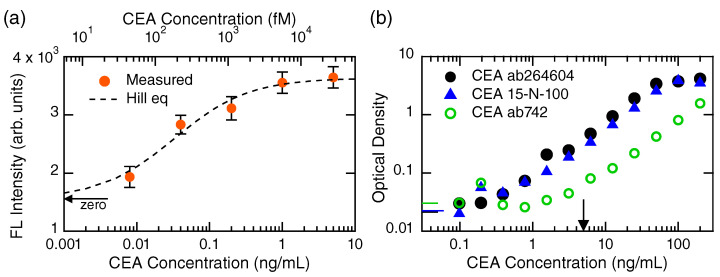
Detection of CEA by the metasurface biosensor and ELISA. (**a**) CEA detection curve in a low concentration range down to 8 pg/mL, measured by the metasurface biosensor. Measured data are shown using orange closed circles with error bars. Dashed curve is a fitted curve by the Hill equation (Equation (1)). (**b**) Detection results for three CEAs (black closed circle: ab264604, Abcam, blue triangle: 15-N-100, Alpha Diagnostic, green open circle: ab742, Abcam) by ELISA, which was conducted using a commercial kit. The vertical axis represents optical density at 450 nm. Three horizontal bars indicate zero levels for the three CEAs; the colors match up the measured data.

A detection curve for CEA in the sample diluent NS buffer is shown in Figure 3b, presented on a semi-log scale. The measured data are shown with orange closed circles associated with error bars. A dashed curve represents a fitted curve using the Hill equation (Equation (1)), which well reproduce the CEA detection data. Thus, the CEA was detected in a scaled manner. A lower concentration range is shown in Appendix (Figure A1a), and the CEA concentration at 0.008 ng/mL was detected in the measurement. From the crossing point of the Hill curve and 3σ level (horizontal bar) in Figure 3b, the limit of detection (LOD) of CEA in this measurement was found to be 0.002 ng/mL (or 11.1 fM). It is to be noted that the detection curve is scaled to the LOD; in other words, the dynamic range covers from 2 pg/mL to 25 ng/mL, exceeding four orders of CEA concentrations. Inset magnifies a concentration range near 0 g/mL on a linear scale.

Figure 3c shows FL signals emitted from the CEA-sandwich bodies in a human serum. The horizontal axis is logarithmic. When we conducted the CEA detection using the serum, the FL-signal level was about five-times lower than that using the NS buffer. It is considered that abundant proteins prevented the CEA and the Abs from forming the sandwich bodies. Accordingly, we conducted a more elaborate FL-signal analysis than that for Figure 3b. In each channel, the FL signal was evaluated in a criterion that intensity more than 3σ from the background level is counted as net signals. This statistical criterion does not output error bar; therefore, the FL signals in Figure 3c are shown only with orange closed circles. A horizontal bar indicates zero-signal level in the measurement. It is to be stressed that the CEA in human serums was detected even at 0.04 ng/mL in a scaled manner; the dynamic range is, at least, 0.04–25 ng/mL. An arrow indicates the clinical diagnosis criterion for CEA, that is, 5 ng/mL [2]. Thus, the metasurface biosensors are capable of detecting CEA in human serums around the diagnosis value.

### 3.2. Coexisting Target Detections

**Figure 4:**
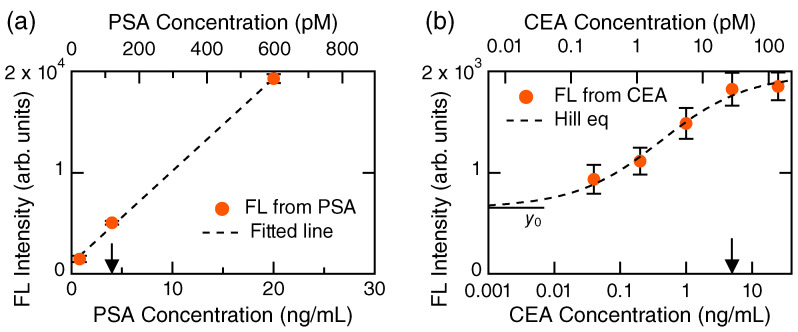
Detection of target cancer markers in coexisting conditions together with other markers. (**a**) PSA detection under a mixture with CEA of 5 ng/mL, presented on a linear scale. FL intensity coming from the PSA is shown using orange circles with error bars. Dashed line: a fitted line. Arrow: the medical criterion value of PSA, 4 ng/mL. (**b**) CEA detection under a mixture with PSA of 4 ng/mL. FL intensity coming from the CEA is shown using orange closed circles with error bars on a semi-log scale. Dashed curve: a fitted curve by the Hill equation (Equation (1)). A black horizontal bar represents the zero level, which is $y_{0}$ in the Hill equation. Arrow: the medical criterion value of CEA, 5 ng/mL.

Detection results under conditions that PSA and CEA coexist are shown in Figure 4. Presentation styles in Figure 4 are similar to those in Figure 2 and Figure 3. We tested two configurations: (i) the target PSA concentrations were 0.8, 4.0, and 20 ng/mL, as shown in Figure 4a, while the CEA concentration was kept at a constant of 5 ng/mL, and (ii) the target CEA concentrations were changed from 0.04 to 25 ng/mL, as shown in Figure 4b, whereas PSA concentration was fixed at 4 ng/mL. In the case (i), CEA was an impeding biomolecule for the target; in (ii), the PSA could impede the detection of target CEA. We note that the clinical criteria for CEA and PSA are 5 and 4 ng/mL, respectively [2].

In both cases, even when the competing cancer markers existed, the target was successfully detected, similarly to the individual detections in Figure 2 and Figure 3. In detail, the FL intensity in the CEA detection became low (Figure 4b), which suggests that the PSA affects the CEA detection; in contrast, there is no definite signature that the CEA affects the PSA detection because the FL intensity was not reduced in Figure 4a, in comparison with that in Figure 2d. Figure 4a is shown on a linear scale and the three data points around the clinical criterion value, 4 ng/mL, were fitted using a line, being well-reproduced (R${}^{2}=0.999$).

## 4. Discussion

Practically, cancer markers must be measured precisely in relation to the clinical criterion values. Here, we discuss the detection results for PSA and CEA using human serums from a practical and critical point of view.

Table 1 lists published information on PSA and CEA detections [6,7,8,9,10,11,12,13,14,15,16,17] and the present results. The dynamic range in Table 1 is defined according to a strict criterion, as is stated in Section 3.1.1. Apart from the claims in the previous reports, the published experimental data were reviewed whether the detection is dynamical, i.e., one concentration is clearly discriminated from the others; for example, if a detected signal at a concentration is overlapped with the other within 1σ, the discrimination is judged to be failure, and the concentration is excluded from the dynamical range in Table 1.

There are mainly two types in the previous reports: one is excessive claims of the dynamical ranges and LODs [7,8,9,10,11,12,13,14,17], and the others are focusing on too low target concentrations in practical senses [6,15]. We here discuss the former cases from a practical viewpoint; the latter does not show any experimental data to support the validity around the clinical criteria. The detections using the electrochemical (EC) methods showed exponential responses, i.e., and most measured data were linearly changed by a factor of 3–5 times and plotted for logarithmic target concentrations, which indicated that narrow signal ranges to the wide target concentrations. Due to such deep sublinear responses, it is generally difficult to discriminate nearest-neighbor concentrations. For example, detection signals at target concentrations of 1 ng/mL and 10 ng/mL cannot be distinguished. This property will be an issue in the practical clinical diagnoses, where definite values should be determined. Thus, it is crucial that signals have a wide dynamic range. It is for this reason that FL detection is considered to be a practically feasible method of detection [33].

For the PSA detections, [8,10,11,13], the dynamic ranges are evaluated to be one or two orders of target concentrations from the strict criterion. In contrast, the present metasurface biosensors provide almost four orders of target concentrations and, furthermore, exhibit robustness for human serums (Figure 2c,e).

The CEA detections based on the EC methods in Table 1 tend to reduce the signals when the target CEA was put in human serums. As an example, the LOD in PBS was claimed to be 0.5 ng/mL, while the CEA detection in human serums was limited to the concentrations at 100 ng/mL and more [6]. A similar reduction in the detection of CEA was reported in an EC method using gold nanoparticles and protein A [34]; the dynamic range was claimed to be from 1 pg/mL to 100 ng/mL; however, the detection range of CEA in rat serum was substantially reduced to a range of 1–50 ng/mL. In the optical sensing [14,16], such heavy reductions were not observed. In this study, although some FL-signal reduction was observed, the reduction was not substantial. Importantly, the metasurface biosensors offer most precise detection among the related reports [6,9,12,14,16,17] and enable parallel detection, even when PSA coexists (Figure 4). It is referred to that optical nanostructured biosensors, such as metasurface biosensors, are extensively explored [35,36,37,38]; so far, better performance and robustness for PSA and CEA than those of the present metasurface biosensors are not found.

## 5. Conclusions

We have tested the detections of two cancer markers, PSA and CEA, using the metasurface biosensors. In the sample diluent buffer and human serums, the target makers were successfully detected in similar manners. The dynamic ranges were almost four orders of target concentrations of PSA and CEA. Furthermore, the metasurface biosensors were hardly affected by the impeding biomolecules in human serums, demonstrating their robustness. Thus, the all-dielectric metasurface biosensors demonstrated highly sensitive and robust detections of the cancer markers.

## 6. Patents

Some of the contents in this article were filed in a Japanese patent (JP2022175611).

## Figures and Tables

**Table 1:** PSA and CEA detections in human serums. EC and NP stand for electrochemical and nanoparticle, respectively. AD-MSB denotes an all-dielectric metasurface biosensor. PlC denotes plasmonic crystal. NS means the sample diluent NS buffer. The dynamic ranges in this Table are not based on the claims in the references, but on the strict criterion described in the text because the ranges were sometimes overestimated.

Target	Method	Feature	Dynamic Range	Buffer	Reference
			(ng/mL)		
PSA	EC	flow on paper	0.063–0.25	Serum	[7]
PSA	EC	MoS${}_{2}$-Au	15–110	Serum	[8]
PSA	EC	Ag NP	2–8	Serum	[10]
PSA	EC	polymer brush-Au NP	1–100	Serum	[11]
PSA	microwell	digital FL	0.002–0.2	Serum	[15]
PSA	AD-MSB	resonance shift	1–8	Serum	[13]
PSA	AD-MSB	FL	0.16–1000	Serum	This work
CEA	EC	aptamer	100–140	Serum	[6]
CEA	EC	polyaniline-Au	1–50	Serum	[9]
CEA	EC	aptamer	5–40	Serum	[12]
CEA	gold PlC	resonance shift	3–18	Serum	[14]
CEA	gold PlC	resonance shift	10–87	Serum	[16]
CEA	gold NP	color change	1–30	Serum	[17]
CEA	AD-MSB	FL	0.002–25	Serum	This work
CEA	ELISA	Absorbance	1–50	NS	Figure A1b

## Appendix A

Figure A1 shows further results of FL detection for CEA using a metasurface biosensor and of CEA detection using a commercial ELISA kit (ab264604, Abcam). Figure A1a shows a lower CEA-concentration range than that is Figure 3a, and indicates that CEA was actually detected at the concentrations of 0.008 ng/mL (or 8 pg/mL). The zero level of the FL measurement is shown with an arrow. Thus, it is confirmed that the dynamic range of the metasurface biosensors for CEA covers from ∼1 pg/mL to 25 ng/mL.

Figure A1b shows a set of ELISA results using the commercial kit, which claims high throughput (i.e., 1.5 h running time) and high sensitivity. We used three CEA targets: block closed circles correspond to CEA, ab264604, Abcam, blue triangles to CEA, CEA15-N-100, Alpha Diagnostic, and green open circles to CEA, ab742, Abcam. The detection profiles of the black closed circle and the blue triangle are similar; the dynamic ranges are estimated to be from 0.63 to 50 ng/mL, based on intersection of the two profiles. In contrast, another profile of the green open circle exhibited a lower response than the others, and the dynamic range covers from 1.25 to 200 ng/mL. These results suggest that the enzyme reaction in ELISA can change for detection targets. Practically, we can conclude that the ELISA kit is effective at 1–50 ng/mL for a given CEA, and that the sub-ng/mL ranges are unreliable.

Additionally, we note that the actual running time for the ELISA was approximately 2 h, due to additional procedures (e.g., washing of a microplate) are inevitable; the runtime falls within a short-time category among various ELISA kits. The metasurface biosensors completed the runtime within 2 h and, moreover, exhibited the one-digit pg/mL detection for CEA in a dynamical manner, which is 100-times more effective than the ELISA kit.
